# Psychological aspects of Recurrent Abdominal Pain Syndrome in children

**Published:** 2015

**Authors:** A Moayedi, F Moayedi

**Affiliations:** * Department of Pediatrics, Clinical Research Development Center of Children Hospital, Hormozgan University of Medical Sciences, Bandar Abbas, Iran; ** Department of Psychiatry, Behavioral and Neurosciences Research Center, Hormozgan University of Medical Sciences, Bandar Abbas, Iran

**Keywords:** recurrent abdominal pain, depression, anxiety, children

## Abstract

**Introduction.** Intermittent visceral distress syndrome is described as “at least three scenes of visceral distress, sufficiently severe to hinder their actions over a time longer than 3 months, continuing from the preceding year”. Organic factors causing abdominal pain are rare, so most of the children with an intermittent visceral distress are designated to have a functional abdominal pain. This study was designed to evaluate psychological problems such as anxiety and distress in children with functional intestinal distress.

**Method.** 120 children (50 boys and 70 girls) with an age range of 5-18 years, who complained of abdominal pain among other things, were included in this cross-sectional case-control study (forty with an organic etiology, 38 diagnosed as RAPS and 42 healthy controls). Revised Children’s Manifest Anxiety Scale (RCMAS) questionnaire and Depression Self-Rated Scale (DSRS) questionnaire were used to determine the level of anxiety. A 28-question General Health Questionnaire (GHQ-28) was also used to investigate the general mental health of their mothers.

**Result.** In the present study, organic and functional etiology of abdominal pain was significantly different with regard to the anxiety score. However, this was not seen as far as depression was concerned. The total GHQ score of mothers was not significantly different between the three groups. ANOVA was used to compare groups.

**Conclusion.** As shown in the present study, that is consistent with most other studies, psychological factors were seen in RAP and need a more in depth investigation to be resolved.

## Introduction

Intermittent visceral injury syndrome (RAPS) was initially defined by Naish and Apley in 1957 as “at least 3 occurrences of visceral pain, sufficiently sharp to hinder their actions beyond a term longer than 3 months, extending from the previous year” [**1**,**2**]. RAP was described in a 10-13 percent of school-aged kids in advanced nations [**2**-**10**]. Organic factors causing abdominal pain are rare, so most of the kids with an intermittent visceral distress were designated to have a functional abdominal pain [**10**].

Exposure to stressful life events was reported in RAPS in previous researches, but there was an inconsistency between their results regarding the presentation of the psychological symptoms. Some case-control researches have an elevated rate of anxiety, depression, and school absence in cases with RAPS compared to the healthy children, but other researches have omitted to show this relationship [**2**-**10**]. The parental role in the functional symptoms of children with RAPS was discussed in the previous studies. A study in London showed that there is a higher likelihood of health complaints in mothers with somatization disorders. Different reactions of these mothers to their children’s abdominal pain are mentioned and considered to have a role in the etiology of RAPS. Also, the genetic transmission and changing the behaviors of children due to the consequences of their experience of pain are mentioned [**11**].

This study was designed to compare psychological problems such as anxiety and depression between children with functional abdominal distress and those with an original etiology and a group of healthy children.

## Materials and methods

120 children (50 boys and 70 girls) with an age range of 5-18 years, who complained of abdominal pain among other things, were included in this cross-sectional case-control study (forty with an organic etiology, 38 diagnosed as RAPS and 42 healthy controls). Healthy controls were among those visited for routine investigations or mild infectious diseases. 

The exclusion criteria in the control group were the following: a history of abdominal pain during the past 3 months, chronic diseases like diabetes mellitus and epilepsy.

The diagnosis of RAPS was based on the pediatrician’s decision according to the ROME criteria. Children in the organic pain group were diagnosed as having peptic ulcers.

The CMAS questionnaire and Depression Self-Rated Scale (DSRS) questionnaire were used to determine the level of anxiety and depression as the two most important etiologies of abdominal pain. A 28-question General Health Questionnaire (GHQ-28) was also used to investigate the general mental health of the mothers. GHQ-28 is a tool that lowers levels and better shows mental health.

The demographic questionnaire included data such as: age, the age at the onset of pain, the numbers of pain episodes in a month and the duration, exacerbating and aggravating factors, the location of pain, other signs and symptoms, family history, family history of psychological problems.

Pain severity was evaluated by an analogue visual scale (VAS) from the children’s and also from their parents’ points of view.

## Results

The mean (± SD) age of these 120 children was 11.52 (± 3.23) years. Female to male ratio in functional, organic and control groups was the following: 25/ 13 (1.92), 23/ 17 (1.35) and 22/ 20 (1.1) (P-value = 0.447). The age at the pain onset was 8.8 (± 3.75) years. The mean episodes of pain were reported to be of 13.26 (± 13.1). The duration of pain was of 2.8 (± 5.8) hours.

The anxiety score was significantly different between the three groups (P-value = 0.000), which was significant between the organic etiology and the functional one (P-value = 0.001) and the functional one and the healthy controls (P-value = 0.000) (**Table 1**).

**Table 1:** The mean value (± SD) of the anxiety score in the 3 groups

Groups	Anxiety score Mean (± SD)
Organic (N = 40)	13.17 (± 4.77)
Functional (N = 38)	17.81 (± 6.41)
Control (N = 42)	11.88 (± 5.93)

The mean (± SD) score of depression was not very significant between the 3 groups (P-value = 0.155) but the difference was significant between the organic etiology group and the control group (P-value = 0.042). 

**Table 2:** The mean value (± SD) of depression score in the 3 groups

Groups	Depression score Mean (± SD)
Organic (N = 40)	11.11 (± 4.8)
Functional (N = 38)	12.42 (± 6.28)
Control (N = 42)	10.07 (± 6.51)

The age at the onset of pain was not significantly different between those with organic abdominal pain (9.52 ± 3.9) and the functional group (7.98 ± 3.5) (P-value = 0.077).

As presented in **Table 3** , there was not a clear distinction between the organic and functional abdominal pain regarding the number of painful episodes (in a month) and the duration of pain (hours). 

**Table 3:** The mean value (± SD) of the number of painful episodes and the duration of pain (hours) in the 2 groups

Groups	Mean (± SD) number of painful episodes in a month	Mean value (± SD) of pain duration (hours)
Organic (N = 40)	15.21 (± 12.6)	1.76 (± 2.24)
Functional (N = 38)	11.25 (± 13.32)	3.85 (± 7.91)
P-value	0.230	0.156

The total GHQ score of mothers was not notably variable among the three teams (P-value = 0.793).

**Table 4:** The mean value (± SD) of the mothers’ GHQ score in the 3 groups

Groups	Mothers’ GHQ score Mean (± SD)
Organic (N = 40)	42.02 (± 8.79)
Functional (N = 38)	44.86 (± 5.5)
Control (N = 42)	43.85 (± 6.19)

The location of pain was significantly different between children with an organic pain and a functional one (P-value = 0.017). In the organic group, it was mostly located in the epigastric area (N = 26), then in the periumblical (N = 16) and hypogastric area (N = 7), but in the functional group it was more often located in the periumblical area (N = 22), epigastric (N = 15) and hypogastric area (N = 5).

The sharpness of the intestinal distress described by the child or the parents was not significantly different between the organic and the functional group (P-value > 0.05).

**Table 5:** The mean value (±SD) of pain severity reported by child and parents

Groups	Pain severity score Mean (± SD) by child	Pain severity score Mean (± SD) by parents
Organic (N = 40)	6.17 (± 1.63)	6.25 (± 1.70)
Functional (N = 38)	5.4 (± 1.85)	5.5 (± 1.92)
P-value	0.052	0.072

## Discussion

The organic and functional etiology of abdominal pain in the present study was significantly different regarding the anxiety score and also the functional score and healthy controls. However, this was not seen regarding depression. The location of pain was significantly different between children with organic pain and functional pain. 

In a study performed in London (2007), Ramchandani et al. enrolled 7128 six-year old children and reported that adolescents with intermittent visceral disorder at age 6 had larger percentages of further visceral disorders, school absence, and anxiety disorders at age seven. Parental stress was the most compatible predictor of consequent adverse consequences for these kids [**2**]. In this research, the impact of maternal stress on the anxiety level of children was not consistent with our results, in which the total GHQ score of mothers was not different between groups. 

Campo et al. (2004) reported that the diagnosis of a psychiatric disorder was significantly more such as the one in RAP patients: stress in 79% and a depressive disorder in 43%, higher stages of stress and depressive signs, sensitive hurt avoidance, and practical impairment than in regulating subjects [**9**]. The present study showed a significant difference between those who suffered from RAP and the control group.

In a study performed in Canada, Kaminsky et al. (2006) enrolled 50 children with RAP (8 to 18) and their mothers. The questionnaire included questions regarding the coping manner, social support, self-efficacy, the locus of control, parental regulation, and emotional regulation, which were answered by both the children and their parents. Their findings suggested that the coping manner, self-efficacy, social support, and maternal regulation were associated with depressive signs in children with RAP [**5**]. As shown in the present study, psychological disorders were seen in RAP and need a more in depth investigation to be resolved.

In a literature review performed by Devanarayana, all the published data from 1958 to 2009 were reviewed, concentrating on the epidemiology, etiology and managing approaches of RAPS in children, the authors concluding that there are functional etiologies causing a recurrent abdominal pain in the majority of children, also suggesting a possible role of the emotional stress, gastrointestinal motility pains, and visceral hypersensitivity in this kind of pain [**1**,**3**]. A relationship between the stressful event and the onset of pain has been shown, the way the child could remember the exact time of the pain starting, which could have been a result of changing school, separation from the parents and birth of sibling [**3**]. However, we did not see these relationships in the present study, but there can always be more possibilities.

## Conclusion

As shown in the present study, the anxiety score was significantly different between the original and the practical etiology of intermittent disorder and the functional one and healthy controls. Children with original intermittent disorder had a larger age of pain. The location of pain was significantly different among children with an original disorder and the ones with a practical one. 

Therefore, the suggestion to have a close observation on school age children and help them in coping with their environmental stress is very important.

**Limitations**

The diagnosis of psychological problems including depression and anxiety was not clinically confirmed in the present study.
